# Pathology of the Landschütz Ascites Tumour

**DOI:** 10.1038/bjc.1964.63

**Published:** 1964-09

**Authors:** A. E. Stuart, A. M. El Hassan

## Abstract

**Images:**


### PATHOLOGY OF THE LANDSCHÜTZ ASCITES TUMOUR

A. E. STUART AND A. M. EL HASSAN

*From the Department of Pathology, University of Edinburgh*

Received for publication May 15, 1964

THE Landschütz transplantable ascites tumour grows rapidly in any strain of mouse despite a minor degree of host resistance. It is not so haemorrhagic as the Ehrlich ascites tumour and is widely used for experimental chemotherapy. Chromosomal studies (Tjio and Levan, 1954) indicate similarity, if not identity, with Ehrlich mammary carcinoma ascites neoplasm. The tumour changes readily from the ascitic to the solid form after treatment with serum or lymphoid cells (Stuart, 1962 ; El Hassan and Stuart, 1963, 1964 ; Stuart and El Hassan, 1963, 1964). Conversion from the ascitic to the solid form alters completely the natural history of the disease. This paper describes the nature of the host resistance and the necropsy findings in mice dying from either the solid or ascitic types of neoplasm.

### MATERIAL AND METHODS

The histology is based on necropsy examination of 800 white mice from a closed colony—M.R.C. endocrinology research unit strain—200 A/Jax Porton strains and 80 $DBA_2$ Porton strain. Solid tumour was obtained from mice treated with serum or lymphoid cells. The tissues were first fixed in 10 per cent formol saline followed by 12–24 hours in saturated corrosive sublimate. Paraffin sections were made and stained with Mayer's haemalum and eosin. Reticulin was demonstrated by an improved silver method (Slidders *et al.*, 1958).

### RESULTS

### *Influence of strain and species differences*

There is a distinct variation in the rate of growth of the Landschütz tumour when $10^5$ cells are inoculated by the intra-peritoneal route into different strains of mouse. Outbred mice develop ascites by the 12th day and survive for approximately 21 days with remarkable constancy. A/Jax strains are more resistant and live for approximately 30 days. $DBA_2$ mice also show a minor degree of resistance. There has been no change in virulence when maintained in outbred mice for 4 years. When first introduced to A/Jax mice minute solid nodules were formed in addition to ascites. After passage for 8 months these nodules were no longer seen at necropsy.

Massive doses will grow progressively in young rats. Smaller doses given subcutaneously grow for a short time and then regress. Regression is accompanied by a vigorous mononuclear cells reaction composed of macrophages and mast cells in the beginning and followed by infiltration with lymphocytes and plasma cells. A subsequent reimplantation of tumour is rejected without a local cellular reaction. In rats the diameter of the tumour does not reflect its true size because a substantial amount of the cellular mass is accounted for by host cell reaction.

*The host response in the mouse*

The spleen increases in weight and by the 10th day is more than twice the normal size. By the 20th day the spleen has substantially decreased in weight.

At necropsy the spleens of some animals are either normal or less than the usual weight although some may be slightly bigger. The reactive spleen as seen 10 days after the inoculation of $10^5$ cells is hypercellular and the malpighian bodies are enlarged. The lymphoid cells of the latter have developed large vesicular nuclei and a moderate amount of finely granular cytoplasm. Mitotic figures are occasionally seen. The red pulp contains foci of immature mononuclear cells with pyronophilic cytoplasm. The endothelial cells are slightly swollen and small pyronophilic globules varying in size from 1 to 3 $\mu$ are frequently present within the lumen of vessels.

Subcutaneous tumours which were not ulcerated show a moderate degree of cellular reaction in the early stages. Fig. 1 shows the cellular reaction between normal skin and an underlying tumour. The dermis is oedematous, congested and contains a mixture of round and spindle cells. A higher magnification (Fig. 2) shows a field selected for its high cellularity. The mononuclear cell response is composed of plasma cells, lymphocytes, monocytes or macrophages and fibroblasts. Although the plasma cells have eccentric nuclei and abundant basophilic cytoplasm, their nuclei are compact and homogeneous. The chromatin pattern of the human plasma cell is not often seen in the mouse. The spindle cells are probably macrophages since we have never seen collagen deposition around this tumour. In the later stages of the disease oedema is still present (Fig. 3) but the inflammatory reaction has been greatly reduced.

The inguinal, thymic, cervical, axillary and abdominal lymph nodes are frequently slightly enlarged. In the late stages of the disease they may contain solid tumour (Fig. 4). Sometimes the gland shows reactive changes (Fig. 5) characterised by increased proportion of immature mononuclear and plasma cells. This is not always so and tumour deposits can be found within lymph nodes without any sign of reactive hyperplasia (Fig. 6).

*Necropsy findings in the untreated animal*

The abdomens of the mice are distended and the peritoneal sac contains 10–15 ml. of ascitic fluid. The fluid contains lipid which will block the pores of a Seitz filter. The abdominal organs may have a thin deposit of fibrin and the spleen often shows acute venous congestion. In A/Jax mice it is often small and hypocellular ; in our outbred strain both size and cellularity vary considerably probably because of an infective agent which we have not identified.

The tumour is always fluid or gelatinous although in one case an A/Jax mouse contained solid tumour. Invasion of organs has never been noted, although sometimes cells which closely resemble or are perhaps identical with ascites tumour cells have been observed in the sinusoids of liver and spleen. These gelatinous masses show extensive necrosis and host cell reaction is absent or minimal. The carcass weight is usually the same or slightly less than the weight of the animal at the time of inoculation of tumour. Anaemia is not conspicuous and develops late. A marked degree of subcutaneous oedema is often present, and is most frequently seen in outbred mice. A conspicuous finding in A/Jax mice is complete atrophy of the thymus. The thymus is often impossible to find at necropsy but sometimes is present, greatly reduced in size with quite prominent lateral para-thymic lymph nodes. The animals die from malnutrition caused by compression of the intestines. Dyspnoea is common and a terminal watery diarrhoea may occur.

*Necropsy findings in animals treated with cells or serum*

Ascites is uncommon and the animals die from complications of a solid infiltrative growth. The tumour cells are 12–14 $\mu$ diameter and have large oval or round nuclei (Fig. 7). The nuclear membrane is thin and distinct. The nuclei contain numerous discrete small basophilic chromatin masses and sometimes round homogeneous eosinophilic bodies are present. They vary in numbers of 1 to 4 and measure about 2 $\mu$ diameter although by coalescence they form quite large asymmetrical masses. The cytoplasm is faintly eosinophilic and rather opaque ; it does not contain granules. The tumour does not form reticulin and Fig. 8 shows preformed reticulin in a paravertebral metastasis. The commonest site for solid tumour is the root of the mesentery where scirrhous tumour encircles the bowel and leads to obstruction. Infiltration of the stomach wall (Fig. 9) may lead to perforation and peritonitis. Sometimes small deposits of ascites cells are present on the mucosal side of the gastric mucosa (Fig. 10). Another common site for tumour growth is the pancreas (Fig. 11) which may be totally replaced by tumour. Sometimes a mass of solid tumour grows in the pelvis and obstructs the ureters with consequent unilateral or bilateral hydronephrosis. Invasion of the kidney is not common but when present the tumour grows around glomeruli (Fig. 12) and tubular necrosis (Fig. 13) may be present.

Solid tumours are often necrotic and may show irregular haematoxyphil bodies which vary in size from 5 to 20 $\mu$ (Fig. 14). They do not stain by Von Kossa's method and are Feulgen positive. The surviving tumour has a paravascular distribution (Fig. 15).

The tumour spreads by lymphatics (Fig. 16 and 17) or direct invasion and we have never seen metastases in the lung. Liver metastases are not common and their distribution suggests direct spread by the portal vein rather than by the systemic blood stream. In such cases the vascular endothelium is activated and small islands of mononuclear cells appear within the sinusoids. Rare complications are hydrothorax due to mediastinal deposits of tumour, obstructive jaundice due to compression of the common bile duct by direct extension of tumour from the root of mesentery to the duodenum and lastly, paralysis. This is caused by invasion of the paravertebral musculature with secondary compression of the spinal cord (Fig. 18). Muscle invaded by ascites tumour cells show swelling and proliferation of the sarcolemma and a cellular reaction composed mainly of lymphocytes and spindle cells (Fig. 19). The vertebral periosteum is thick and hyperplastic in the vicinity of tumour (Fig. 20).

### DISCUSSION

The Landschütz tumour arose spontaneously in a strain of Rockefellar white mice and was subsequently propagated by Landschütz in outbred animals and described as a reticulum cell sarcoma (Tjio and Levan, 1954). These authors made a careful and detailed study of the chromosomes of this tumour. They noted the resemblance of the Ehrlich mammary carcinoma and thought the two tumours were related. Our histological observations suggest that this tumour is a carcinoma because it spreads by lymphatics, and when present in lymph nodes it forms discrete masses. It grows in solid cords or sheets, is well vascularised and the tumour cells radiate from well formed vessels. The absence of pericellular reticulin does not help and cytologically we cannot confidently distinguish between reticulum cell sarcoma and carcinoma, although perhaps the rather coarse chromatin pattern favours the latter diagnosis.

The host reaction can be conveniently studied in the dermal response to subcutaneous tumour or the splenic reaction invoked by the ascitic growth. It is noteworthy that in the late stages of tumour growth the dermal response is diminished and in A/Jax mice the spleen is often reduced in size. Thus necropsy examination adequately fails to reveal a previous state of host reaction. This reaction could be due to either bacterial or viral contamination, especially since it is well known that " passenger " viruses may multiply in ascites tumour cells. The nucleoli seen in tissue sections have an unusual morphology and may in fact be viral inclusions. However, cultures of ascitic fluid were sterile and a Seitz filtrate of tumour fluid has so far failed to produce splenomegaly. These bacteriological investigations are incomplete, and although so far negative we think the interpretation of splenomegaly following inoculation of a transplantable tumour remains hazardous. Stansley, Ramsay and Neilson (1962) and Ansari, Neilson and Stansley (1963) have demonstrated a "transmissible spleen weight increase factor " of mice. This agent can be transmitted by homogenates of spleen but the pathology and incubation times are different from the splenomegaly described here. " Splenomegaly " or enlargement of lymph nodes is well recognised in rodents bearing tumour (Parsons, Gullard and Barker, 1947 ; Antopol, Claubach and Graff, 1954 ; Rodriguez and Cerecedo, 1955 ; Old *et al.*, 1960 ; Woodruff

### EXPLANATION OF PLATES

FIG. 1.—Subcutaneous tumour. H. and E. ×90.
FIG. 2.—Higher magnification of Fig. 1. H. and E. ×345.
FIG. 3.—Subcutaneous tumour. H. and E. ×210.
FIG. 4.—Metastatic tumour in lymph node. H. and E. ×135.
FIG. 5.—Higher magnification of lymphoid tissue in Fig. 4. H. and E. ×600.
FIG. 6.—Metastatic tumour in lymph node. Note absence of reactive changes. H. and E. ×270.
FIG. 7.—Solid ascites tumour. H. and E. ×270.
FIG. 8.—Tumour cells growing in preformed reticulin of vertebral muscle. Note absence of pericellular reticulin. Silver impregnation. ×270.
FIG. 9.—Solid tumour infiltrating submucosa and muscle layers of stomach. H. and E. ×78.
FIG. 10.—Deposits of tumour in mucosa of stomach. H. and E. ×330.
FIG. 11.—Tumour infiltration of pancreas. H. and E. ×135.
FIG. 12.—Periglomerular growth of tumour. H. and E. ×270.
FIG. 13.—Tubular necrosis of kindey. H. and E. ×180.
FIG. 14.—Haemotoxyphil bodies in necrotic tumour. H. and E. ×400.
FIG. 15.—Paravascular distribution of tumour cells. H. and E. ×40.
FIG. 16.—Tumour in lymphatic. H. and E. ×320.
FIG. 17.—Perineural spread of tumour. H. and E. ×180.
FIG. 18.—The bottom right-hand corner shows a dark staining tumour mass in paravertebral soft tissue. From a paraplegic mouse. H. and E. ×20.
FIG. 19.—Invasion of muscle with minimal reactive change. H. and E. ×320.
FIG. 20.—Periosteal reaction to tumour. H. and E. ×320.

1 2 3 4 5 6

Stuart and El Hassan

7 8 9 10 11 12

Stuart and El Hassan.

13

14

15

16

Stuart and El Hassan.

17

18

19

20

Stuart and El Hassan.

and Symes, 1962). Albert, Johnson and Pinkus (1954) investigated the effect of both transplanted and spontaneous mammary gland carcinomas on lymph nodes and spleen. Homologous transplants increased both weight and the uptake of radioactive phosphorus. Thus at the present time it seems likely that an antigenic difference exists between tumour and host. This is supported by our finding that the tumour inhibiting activity of spleens removed 10 days after implantation of tumour is greater than those taken at 21 days when the weight of the spleen has diminished (unpublished observation). The finding of different growth rates of the tumour in different strains of host is consistent with the view that the antigen of the ascites cell is physiological and not a pathological mutant which would evoke a similar response from different hosts. Further support for the antigenicity of this ascites tumour is derived from our observation that it may be greatly enhanced under certain conditions by transfer of either lymphoid cells or serum.

The necropsy findings in animals incompletely treated with either lymphoid cells or serum show they have died from the local complications of solid tumour such as intestinal obstruction, peritonitis, pancreatic insufficiency, uraemia, jaundice, paralysis and widespread metastases. This agglutination of the ascites cells means that the animals live very much longer than the untreated controls and that the usual parameter of tumour growth such as time of onset of ascites and survival time are both inaccurate and misleading. The persistent growth of the tumour in the solid form long after exposure to an immunological constraint is difficult to explain but teleologically represents a defence reaction by the neoplasm against an unfavourable environment.

### SUMMARY

The Landschütz ascites tumour may be converted to the solid form by serum or lymphoid cells. It behaves like a carcinoma and induces a host response as judged by round cell infiltration and splenomegaly. This host reaction diminishes in the later stages of tumour growth. Treated animals die from the local complications of solid tumour such as intestinal obstruction, peritonitis, pancreatic insufficiency, uraemia, jaundice, paralysis and widespread metastases. This agglutination of ascites cells means that the animals survive much longer than untreated controls.

We are indebted to Professor G. L. Montgomery for his encouragement and help with the manuscript.

A. E. Stuart gratefully acknowledges a research fellowship from the Scottish Hospital Endowment Research Trust and a generous grant from the British Empire Cancer Campaign for Research.

A. M. El Hassan is in receipt of a scholarship from the University of Khartoum.

### REFERENCES

ALBERT, S., JOHNSON, R. M. AND PINKUS, H.—(1954) *Cancer Res.*, **14**, 710, 714.

ANSARI, K. A., NEILSON, C. F. AND STANSLEY, P. G.—(1963) *Exp. molec. Path.*, **2**, 61.

ANTOPOL, W., CLAUBACH, S. AND GRAFF, S.—(1954) *Proc. Amer. Ass. Cancer Res.*, **1**, 2.

EL HASSAN, A. M. AND STUART, A. E.—(1963) *Lancet*, ii, 496.—(1964) *J. Path. Bact.* (in press).

OLD, L. J., CLARKE, D. A., BENACERAFF, B. AND GOLDSMITH, M.—(1960) *Ann. N.Y. Acad. Sci.*, **88**, 265.
PARSONS, L. D., GULLARD, J. M. AND BARKER, G. R.—(1947) *Symp. Soc. exp. Biol.*, No. 1, 179.
RODRIGUEZ, N. M. AND CERECEDO, L. R.—(1955) *Growth*, **19**, 31.
SLIDDERS, W., FRASER, D. S. and LENDRUM, A. C.—(1958) *J. Path. Bact.*, **75**, 478.
STANSLEY, P. G., RAMSAY, D. S. AND NEILSON, C. F.—(1962) *Proc. Soc. exp. Biol. N.Y.*, **109**, 264.
STUART, A. E.—(1962) *Lancet*, ii, 180.
*Idem* AND EL HASSAN, A. M.—(1963) Third International Congress on Chemo-therapy, Stuttgart, E15. (G. Thieme.)—(1964) *Lancet*, i, 913.
SLIDDERS, W., FRASER, D. S. AND LENDRUM, A. C.—(1958) *J. Path. Bact.*, **75**, 478.
TJIO, J. H. AND LEVAN, A.—(1954) *Act. Univ. lund*, **50**, 1.
WOODRUFF, M. F. A. AND SYMES, M. O.—(1962) *Brit. J. Cancer*, **16**, 120.


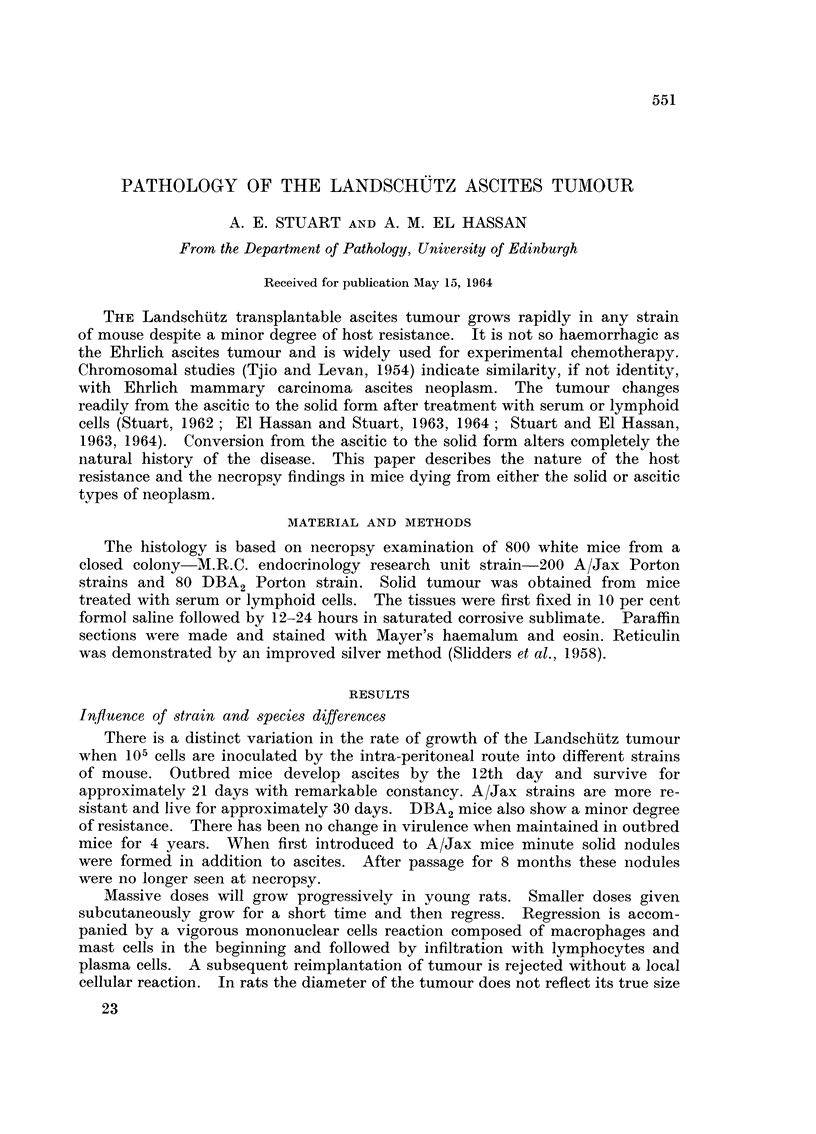

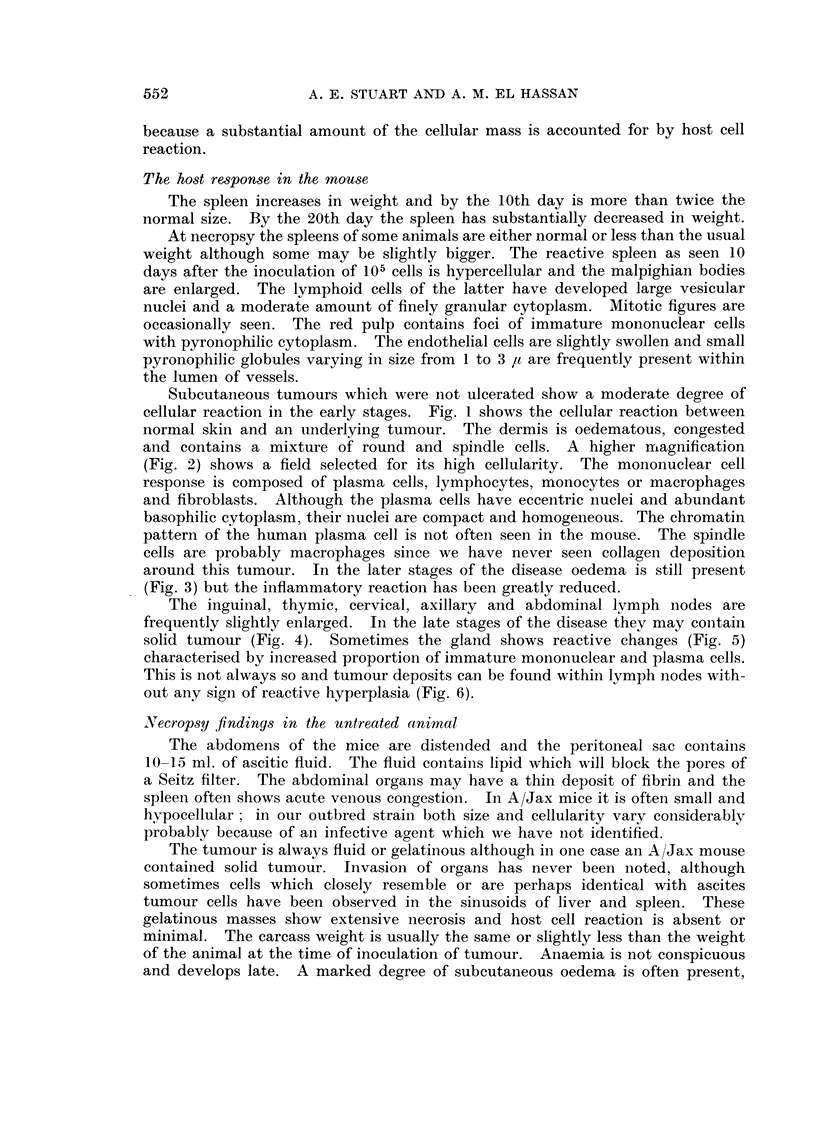

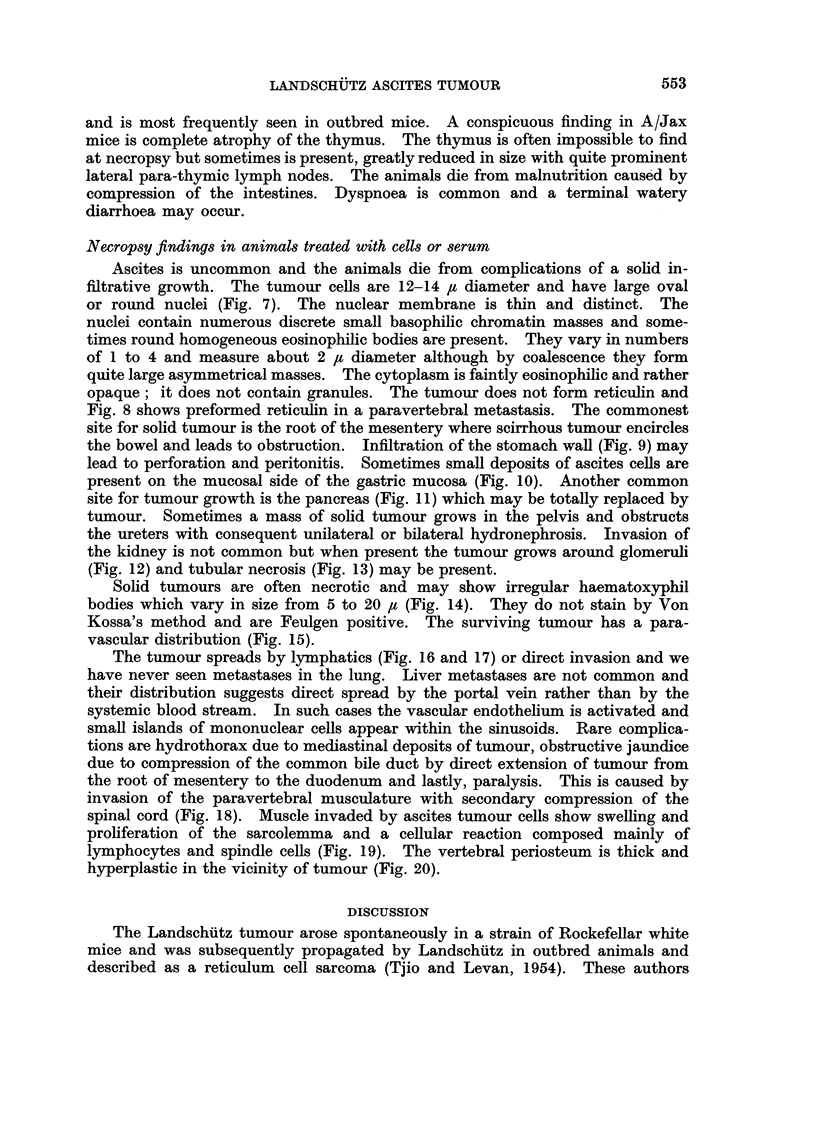

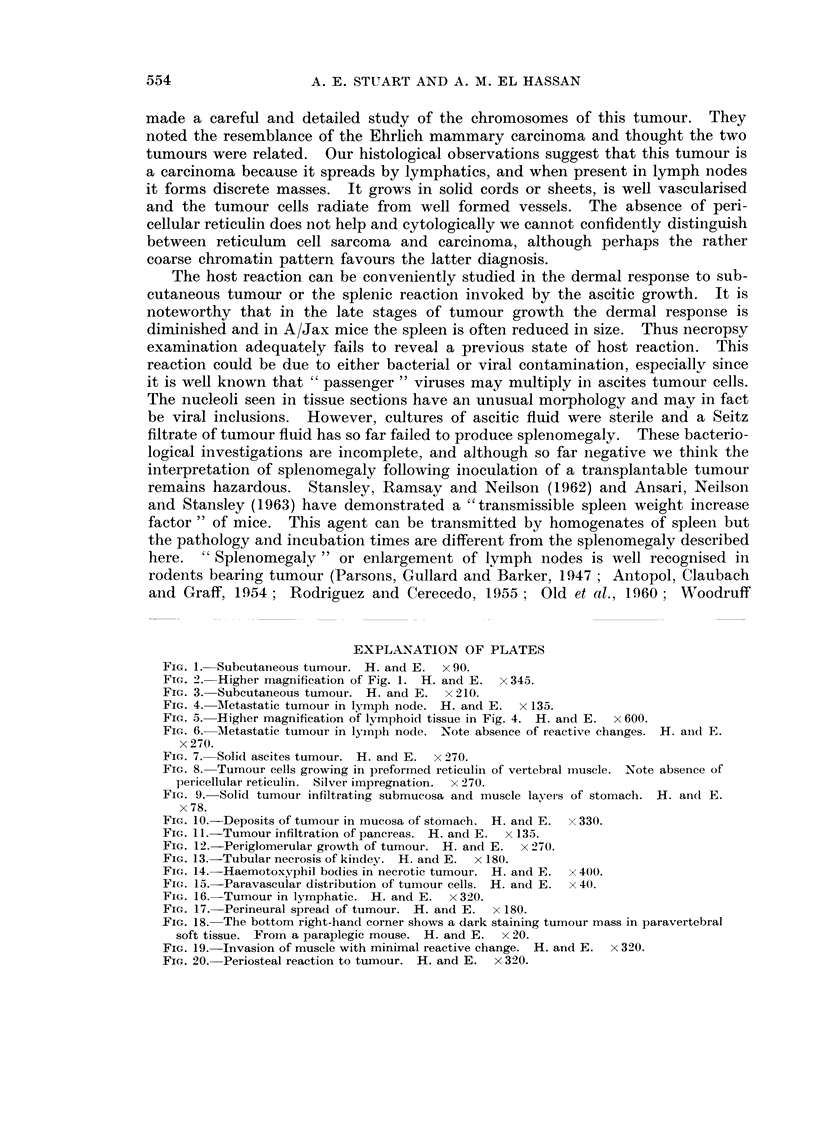

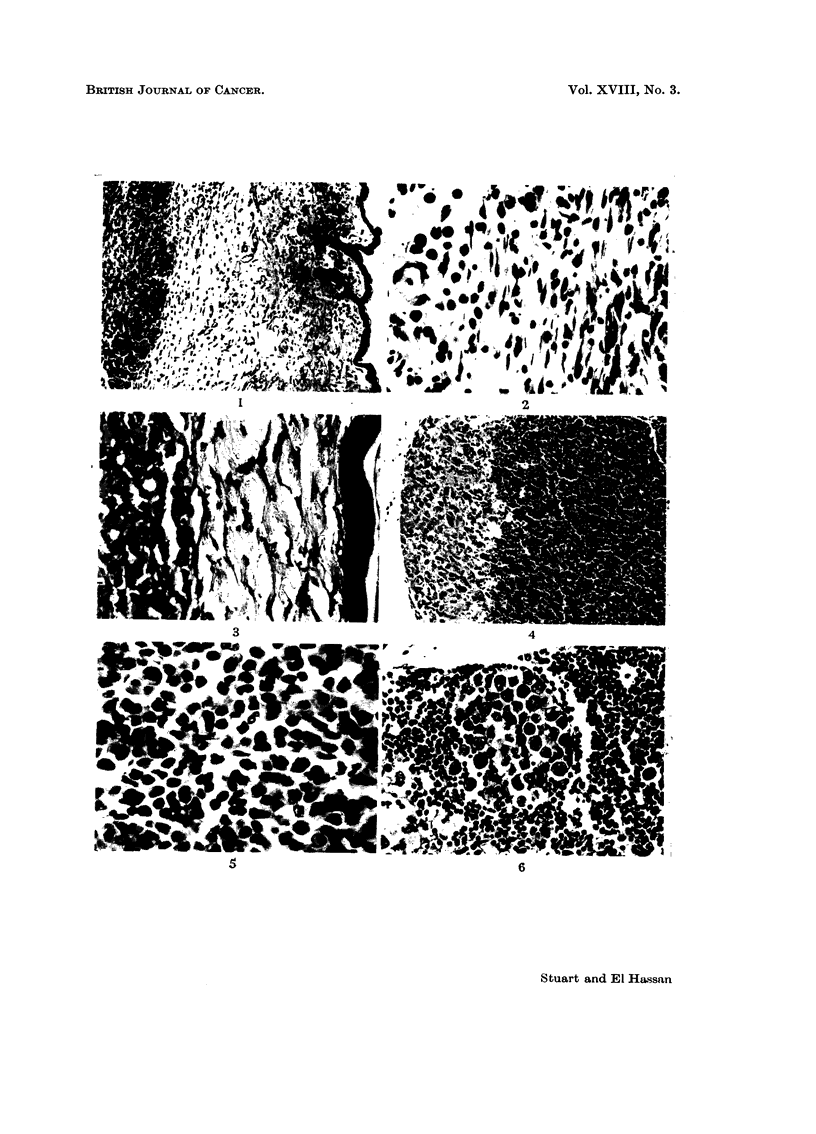

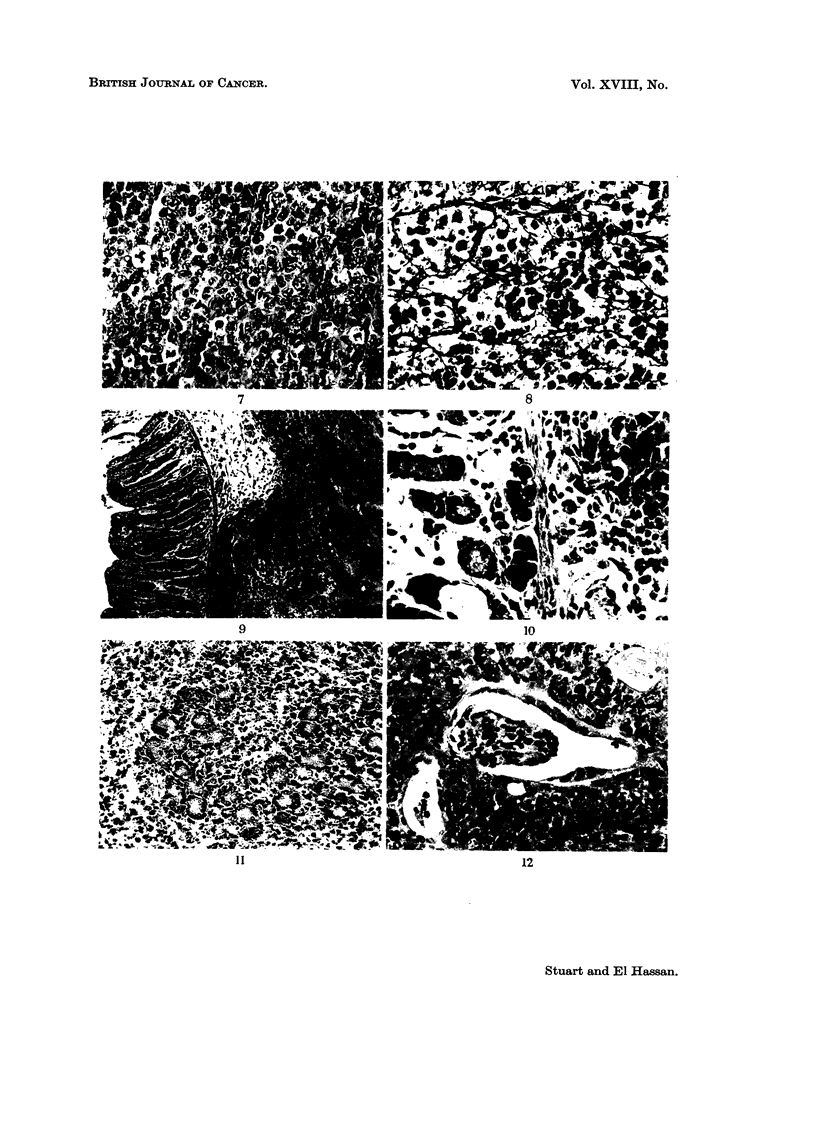

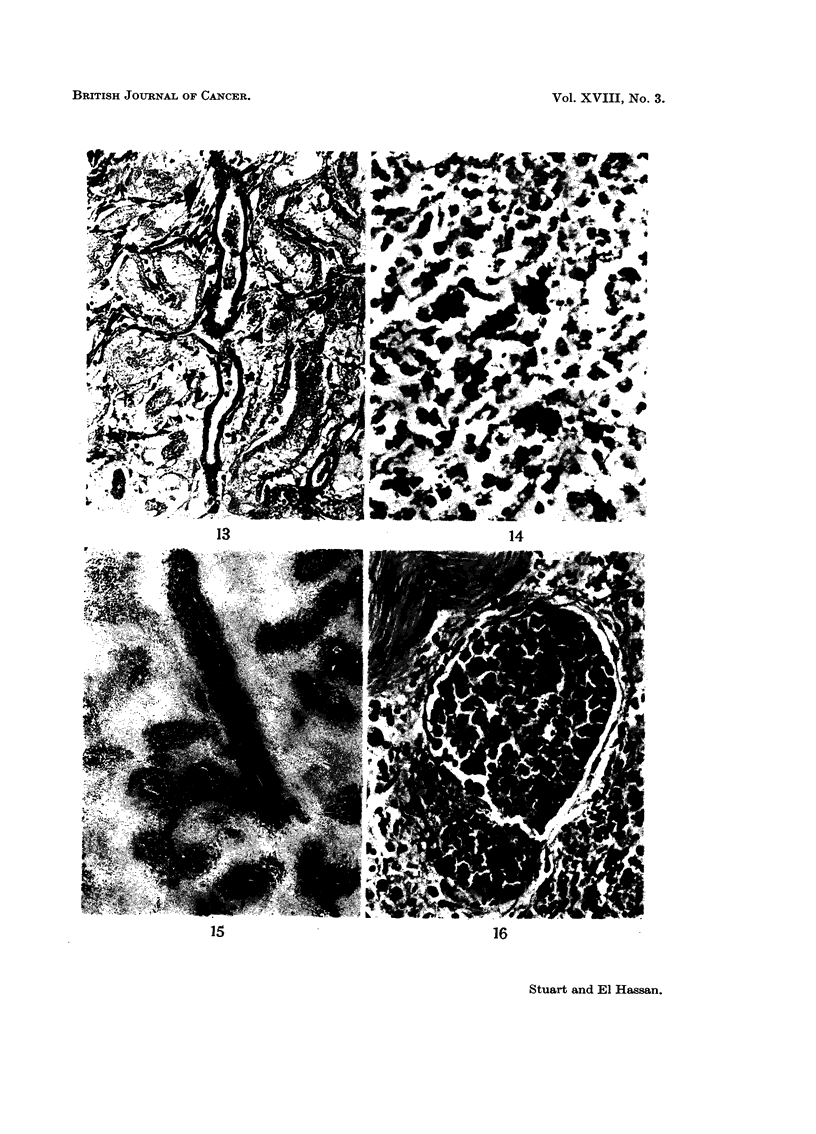

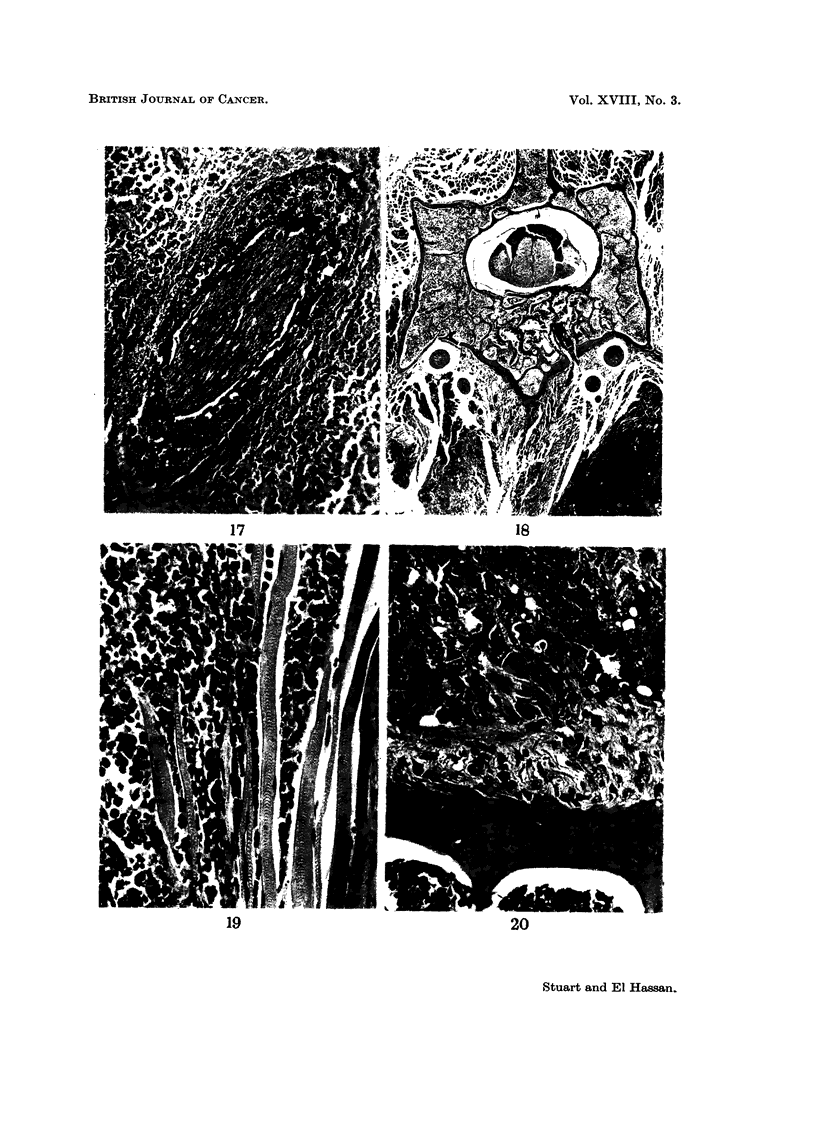

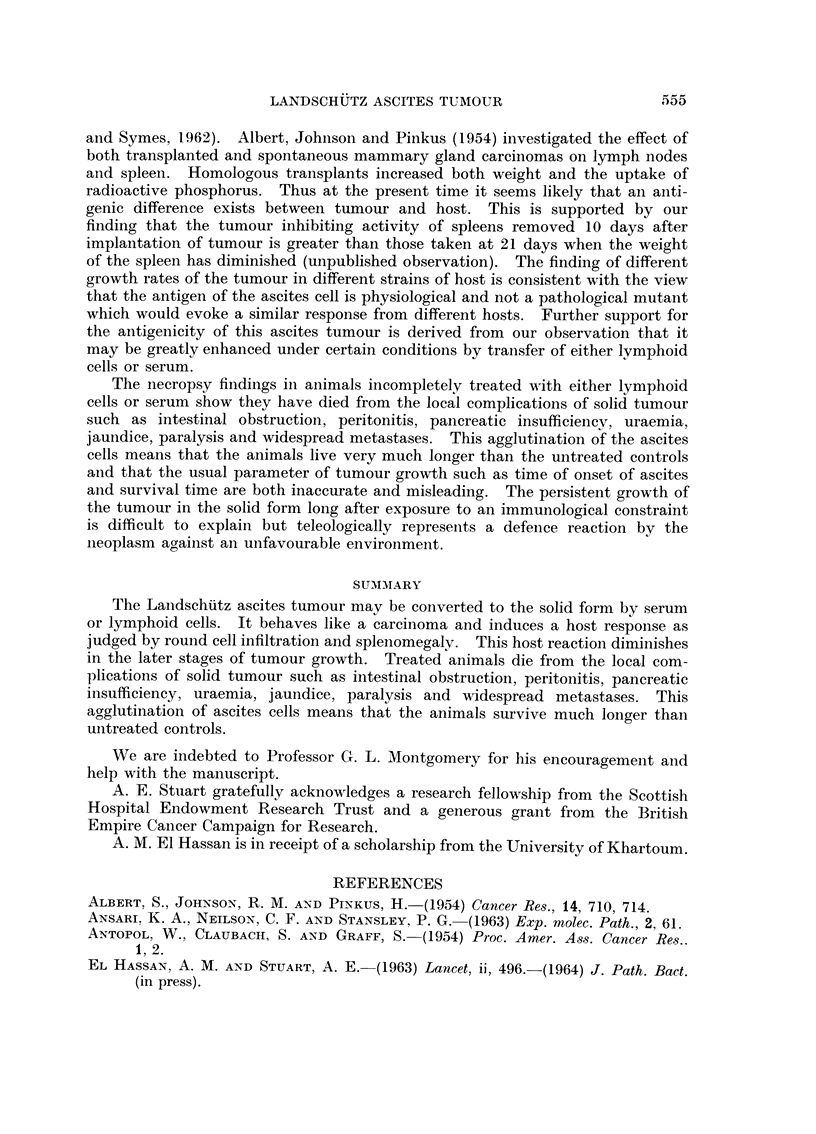

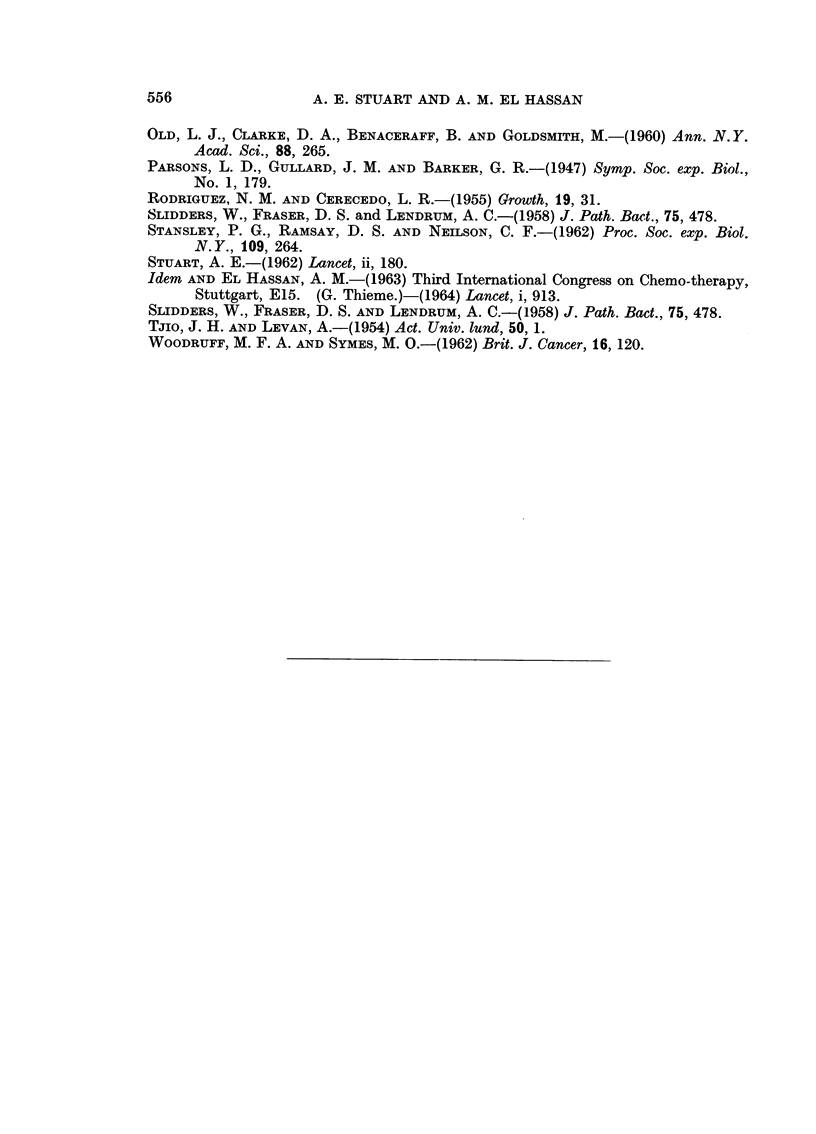


## References

[OCR_00365] ALBERT S., JOHNSON R. M., PINKUS H. (1954). The effect of transplanted and spontaneous mouse mammary gland carcinoma on lymph nodes.. Cancer Res.

[OCR_00401] SLIDDERS W., FRASER D. S., LENDRUM A. C. (1958). Silver impregnation of reticulin.. J Pathol Bacteriol.

[OCR_00391] STANSLY P. G., RAMSEY D. S., NEILSON C. F. (1962). A transmissible spleen weight increase factor (SWIF) of mice.. Proc Soc Exp Biol Med.

[OCR_00395] STUART A. E. (1962). The cytotoxic effect of heterologous lymphoid cells.. Lancet.

[OCR_00404] WOODRUFF M. F., SYMES M. O. (1962). The significance of splenomegaly in tumour-bearing mice.. Br J Cancer.

